# “Soldier's Heart”: A Genetic Basis for Elevated Cardiovascular Disease Risk Associated with Post-traumatic Stress Disorder

**DOI:** 10.3389/fnmol.2016.00087

**Published:** 2016-09-23

**Authors:** Harvey B. Pollard, Chittari Shivakumar, Joshua Starr, Ofer Eidelman, David M. Jacobowitz, Clifton L. Dalgard, Meera Srivastava, Matthew D. Wilkerson, Murray B. Stein, Robert J. Ursano

**Affiliations:** ^1^Department of Anatomy, Physiology and Genetics, Uniformed Services University School of Medicine, Uniformed Services University of the Health SciencesBethesda, MD, USA; ^2^Collaborative Health Initiative Research Program, Uniformed Services University of the Health SciencesBethesda, MD, USA; ^3^Department of Psychiatry, University of California, San DiegoSan Diego, CA, USA; ^4^Department of Psychiatry Uniformed Services University School of Medicine, Uniformed Services University of the Health SciencesBethesda, MD, USA; ^5^Center for the Study of Traumatic stress, Uniformed Services University of the Health SciencesBethesda, MD, USA

**Keywords:** post traumatic stress disorder, cardiovascular disease, Type 2 diabetes mellitus, candidate gene

## Abstract

“Soldier's Heart,” is an American Civil War term linking post-traumatic stress disorder (PTSD) with increased propensity for cardiovascular disease (CVD). We have hypothesized that there might be a quantifiable genetic basis for this linkage. To test this hypothesis we identified a comprehensive set of candidate risk genes for PTSD, and tested whether any were also independent risk genes for CVD. A functional analysis algorithm was used to identify associated signaling networks. We identified 106 PTSD studies that report one or more polymorphic variants in 87 candidate genes in 83,463 subjects and controls. The top upstream drivers for these PTSD risk genes are predicted to be the glucocorticoid receptor (NR3C1) and Tumor Necrosis Factor alpha (TNFA). We find that 37 of the PTSD candidate risk genes are also candidate independent risk genes for CVD. The association between PTSD and CVD is significant by Fisher's Exact Test (*P* = 3 × 10^−54^). We also find 15 PTSD risk genes that are independently associated with Type 2 Diabetes Mellitus (T2DM; also significant by Fisher's Exact Test (*P* = 1.8 × 10^−16^). Our findings offer quantitative evidence for a genetic link between post-traumatic stress and cardiovascular disease, Computationally, the common mechanism for this linkage between PTSD and CVD is innate immunity and NFκB-mediated inflammation.

## Introduction

Soon after the end of the American Civil War, Dr. Jacob Mendez Da Costa, a Philadelphia physician, reported evidence linking what we now term post-traumatic stress disorder (PTSD) with increased risk for cardiovascular disease (CVD; Wooley, 1982). Based on analysis of *ca*. 300 soldiers in a dedicated hospital in wartime Philadelphia, Da Costa's report may have been the first ever example of a modern “big data” clinical study. Da Costa termed the relationship “soldiers heart ” or “irritable heart,” More recently, this relationship has been described in different groups of combat veterans and civilians with PTSD (Cwikel et al., 1997; Paulus et al., 2013; Sidney, 2013; Turner et al., 2013; Wentworth et al., 2013; Beristianos et al., 2014; Roy et al., 2015). Until very recently, there was no biological evidence to link PTSD to CVD. However, the possibility of a genetic mechanism for the link was recently raised by twin studies from the Viet Nam Era Twin (VET) Registry (Vaccarino et al., 2013). This study showed that if both twins had PTSD, the risk of CVD was doubled in both twins. Strikingly, the increased risk was unrelated to smoking, blood lipids, obesity or lack of exercise. By contrast, no significantly increased *familial* risk for Type II diabetes could be found in the same cohort (Vaccarino et al., 2014). Together, these epidemiological and experimental data suggest that a hitherto unknown genetic mechanism might be responsible for the link between PTSD and CVD.

However, to determine what common genetic mechanisms might be responsible, a systems biology approach must be deployed. Alternative approaches include Genome Wide Association Studies (GWAS), Whole Exome Sequencing (WES), Whole Genome Sequencing (WGS), and Candidate Gene Analysis (CGA). However, recent GWAS for PTSD have provided limited evidence of association for specific loci (Andero et al., 2013; Logue et al., 2013; Xie et al., 2013; Stein et al., 2016). GWAS studies are based on finding a significant association between disease and up to 500,000 single nucleotide polymorphisms (SNPs) which are randomly distributed in the 3.6 Billion base human genome. Thus, a 500,000 SNP survey hopes to find a genome-wide disease association by targeting just 0.16% of the genome. If successful in discovering a significant association for a SNP, or set of SNPs, with disease, the next step is to look for a gene near the SNP and *impute* that nearby gene for association with the disease. Significance is based on the Bonferroni correction for multiple comparisons. A significant *p*-value based on this correction is computed by dividing the standard *p*-value for significance (e.g., 0.05) by the number of comparisons (*viz*, corrected *p* = 0.05/500,000 = 1 EXP-7; EXP-7 also means 1 × 10^−7^). Thus, a *p*-value equal to or less than 10^−7^ would be nominally significant.

Thus, in the largest study to date in a military ArmySTARRS population, *ANKRD55*, on chromosome 5, was imputed from a significant association (*p* = 2.34 EXP-8) at rs159572. This SNP (single nucleotide polymorphism) was located within an intron of the *ANKRD55* gene in African Americans only (Stein et al., 2016). By contrast, in the same study, a significant association for European-Americans was imputed for *ZNF626* on chromosome 19 (*p* = 4.59 EXP-8). Furthermore, no significant associations were observed for Latinos or in any of the transethnic meta-analyses. In a different study on a smaller group from the military, with non-hispanic and African American subcohorts, the retinoid-related orphan receptor alpha (RORA) was imputed from a significant association (*p* = 5 EXP-8) with the SNP at rs8042149 (Logue et al., 2013). Importantly, this SNP was also located *within* the RORA gene itself. However, in spite of a similar African American cohort, the SNP at rs159572, imputing *ANKRD55*, was not found. Finally, Xie et al. (2013), studying a racially mixed civilian cohort, identified a significant SNP (*p* = 3.97EXP-8) at rs406001 for the European American population only. However, no candidate gene could be imputed from this SNP. Thus, these three most recent and best powered studies do not replicate each other, even though the individual associations from these studies are significant, and the identifying SNPs for two of the differently imputed genes are located *within* their respective gene sequences. Furthermore, the systems biology approach cannot be deployed without a *pattern* of PTSD-associated SNPs, to compare with parallel data from CVD. We therefore conclude that it is not yet possible to deduce *common* candidate risk genes for PTSD from GWAS data alone.

However, WES and WGS are emerging technologies which have not yet been deployed for PTSD. Therefore, to test the hypothesis of a genetic basis for the connection between PTSD and CVD, we are left presently only with the Candidate Gene approach. The Candidate Gene approach depends on collecting and analyzing genes tested for disease association based on a hypothesis-driven approach by investigators, based on knowledge or intuition regarding the disease of interest. Although this strategy is potentially conflicted by investigator bias, the bias can be diluted out by a large number of studies, and has been used as a bootstrap to enhance focused genomic analysis of complex traits. Recent examples include asthma (Michel et al., 2010) and obesity (Kim et al., 2012). Therefore, as a first step toward the candidate gene approach, we identified 106 PTSD studies that report one or more polymorphic variants in 87 candidate PTSD risk genes in 83,463 subjects and controls. We then asked (1) whether any of the PTSD risk genes overlapped with an equivalent list of independent risk genes for either CVD or T2DM: there were 36 and 15 respectively; (2) whether the associations were significant: they were; and (3) whether an informatics-based analysis might identify shared signaling pathways; they did, emphasizing the NFκB complex and downstream proinflammatory signaling. We conclude that these data provide mechanistic evidence of a genetic link between susceptibility to both PTSD, cardiovascular disease, and to a lesser extent Type 2 Diabetes Mellitus. However, we suggest that validation of this conclusion will necessarily depend on future WGS of well-phenotyped PTSD populations.

## Results

### Identification of candidate genes associated with increased risk for PTSD

The results of our survey of *candidate* risk genes reported for PTSD are summarized alphabetically in Supplementary Table 1. They include the results of 106 studies reporting one or more polymorphic variants in 87 genes in 83,463 subjects and controls. We employed a manual curation approach to this analysis, in order to ensure that the claim in each study was fully validated by the data presented. No meta-data analyses were performed. Immediate inspection of Supplementary Table 1 indicates that at least 13 of these genes have been reported by more than one group of investigators. These include adenylate cyclase 8 (brain) (ADCY8); adenylate cyclase activating polypeptide 1 (pituitary) receptor type 1 (ADCYAP1R1); brain derived neurotropic factor (BNDF); catechol-O-methyltransferase COMT); chemokine (C-X-C) ligand 8 (IL-8); dopamine receptor 2(DRD2); FK506 binding protein 5 (FKBP5); interleukin 1beta (IL1B); glucocorticoid receptor (NR3C1); regulator of G protein signaling 2 (RGS2); neuronal/epithelial high affinity glutamate transporter (SLC1A1); dopamine transporter (SLC6A3); serotonin transporter (SLC6A4); and tryptophan hydroxylase 2 (TPH2). These genes thus survive at least one conventional candidate gene test of reproducibility, while the others on the list remain to be tested in more than one venue.

However, it is yet to be determined whether the variants for these genes, or others on the list, effect increased risk for PTSD for only some populations. For example, some of these reported PTSD risk genes have been specifically associated in their respective literatures with sub-populations of varying ethnicity and genders. Examples include Tryptophan Hydroxylase 1 (TPRH; found in Spitak earthquake survivors), 5-Hydroxytryptamine Receptor 2A (5-HT2A: only reported for an African American population; possibly only in females); Adenylate Cyclase Activating Polypeptide 1 (Pituitary) (Receptor Type 1 (PACAPR: females but not males); Corticotropin Releasing Hormone Receptor 1 (CRHR1: post hurricane survivors); Regulator of G-Protein signaling 2 (RGS2, GOS8: post hurricane survivors); Steroid-5 Alpha Reductase, Alpha Polypeptide 2 (SRD5A2: males but not females); Stathmin 1 (SMN: females only, in the Wenchen earthquake); WW and C2 Domain Containing 1 (KIBRA, PPP1R169: in an African population); Tolloid-like-1 (TLL1: identified in European/Hispanic American but not African American combat veterans); and Phosphoribosyltransferase Domain Containing 1 (PRTFDC1: applicable to multiple ethnicities, including Native American). Thus, there may be ethnic and gender-dependent heterogeneity in the complete list. However, this potential problem cannot be known with certainty, given the variable numbers of subjects and controls in each cohort, the consequent questions of statistical power, and the limitations of previously available genetic tools.

### Signaling pathways and networks associated with PTSD susceptibility genes

It was therefore possible that genetic risk for PTSD susceptibility might have such considerable allelic variation that considering the entire group for analysis might be overwhelmed by this heterogeneity. However, network science, especially for the primate brain, can provide strong inference suggestions as to function (Dalgard et al., 2015; Mears and Pollard, 2016). Analogous to airline destination maps which describe major airport hubs and feeder connections to minor airports, pathway analysis software can use interaction data mined out from the literature to discern such relationships among genes and proteins. The relationships include disease-relevant pathways, predicted causal (upstream) drivers, and calculations of statistical significance based on the number of genes known to be in a specific pathway relative to the number of genes in the experimental sample. Therefore, to identify PTSD-specific signaling pathways and calculate significance, we used the Ingenuity Pathway Analysis (IPA) software. IPA is one of the more widely used proprietary recipes, or algorithms, for this purpose.

**Figure 1:**
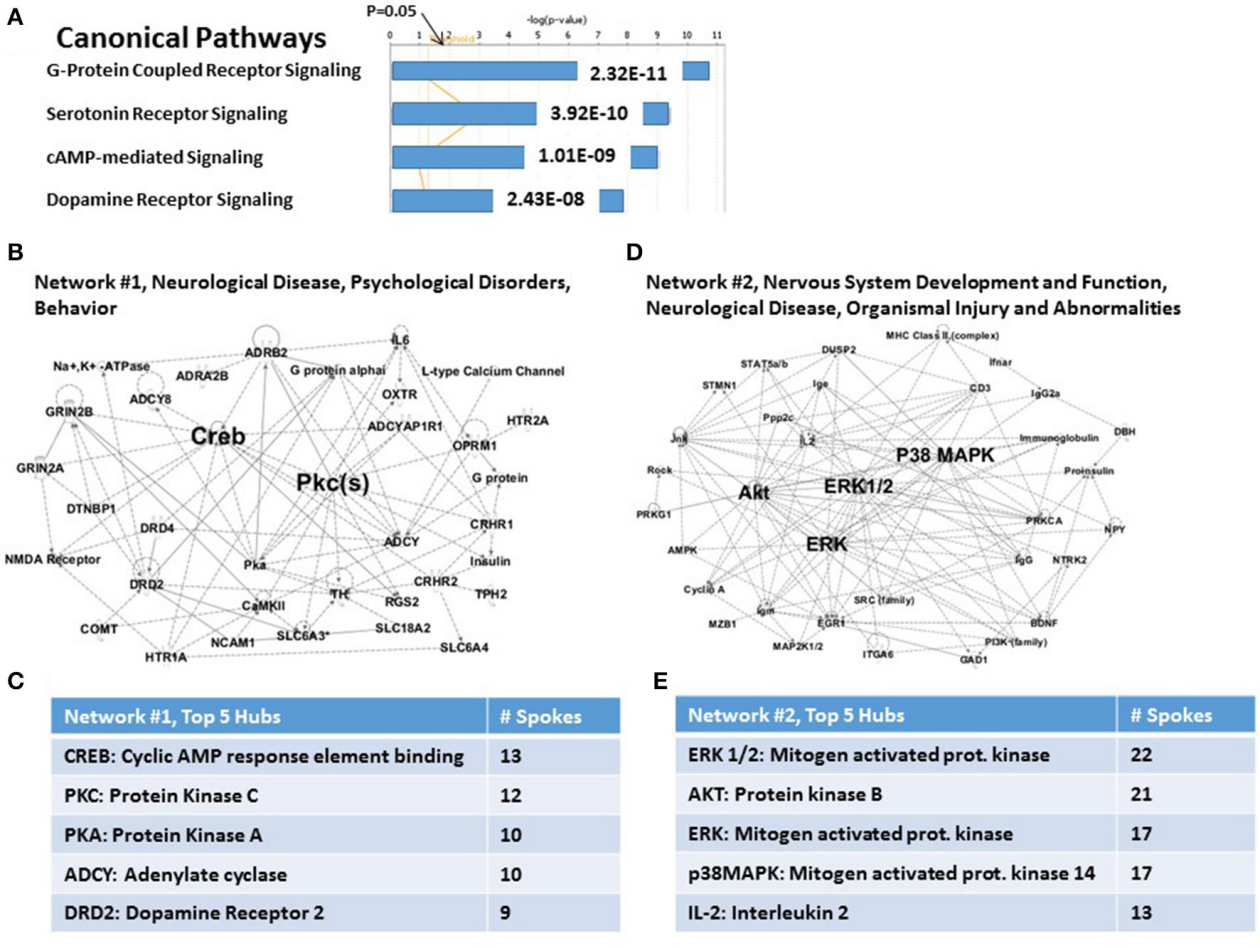
**Functional relationships among 83 candidate PTSD genes. (A)** Top canonical pathways. Two top signaling pathways are G-Protein Coupled Receptor and Serotonin Receptor signaling. **(B)** Associated network Function #1. CREB and PKC are top hubs in this network. **(C)** *Inset*: Network #1, top 5 hubs. **(D)** Associated network Function #2. ERK1/2, AKT, ERK, and p38MAPK are top hubs in this network. **(E)** *Inset*: Network #2, top 5 hubs.

The IPA analysis software used to analyze these 83 candidate PTSD risk genes as a group, allowed us to identify a limited number of highly significant, biologically relevant, Canonical Pathways and Associated Networks. As shown in Figure 1A, the 4 top highly significant Canonical Pathways include G-Protein Coupled Receptor Signaling (*P* = 2.32E-11), Serotonin Receptor Signaling (*P* = 3.92E-11); cAMP-mediated signaling (*P* = 1.01E-09); and Dopamine Receptor Signaling (*P* = 2.43E-08). As shown in Figure 1B, the algorithm also identified “Neurological Disease…” (Network #1) and “Nervous System Development and Function…” (Network #2) as the principal predicted functional consequences when all 83 genes were considered together. These data are thus generally consistent with what might be expected from an analysis of a brain-centric psychiatric disease like PTSD.

For the case of *Network #1* (Figure 1B), 22 of 26 nodes in this graph are genes from the complete set of 83. Figure 1C shows the principal hubs which integrate these genes, including CREB (cAMP response element binding protein: 13 of 26 nodes) and Protein Kinase C (12 of 26 nodes). The hubs shown are in descending order of connectedness, and thus possibly in descending order of importance for control of PTSD susceptibility. For the case of *Network #2* (Figure 1D), 11 of 27 nodes in this graph are genes from the complete set of 83. Figure 1E shows the principal hubs which integrate these genes, including ERK1/2 (22 of 27 nodes) and AKT (Protein Kinase B: 21 of 27 nodes). Importantly, nodes for genes or functions that not in the original set of 83 genes are not necessarily “false positives”; rather they have traditionally been viewed as representing candidate genes or functions for further study. *Network #2*, for example, includes proinsulin, PI3K, AMPK and MHC Class II, genes or functions which may become of interest in a later part of this paper. However, comprehensively, the two networks together represent 33 genes (*viz*., *ca*. 40% of the total).

**Figure 2:**
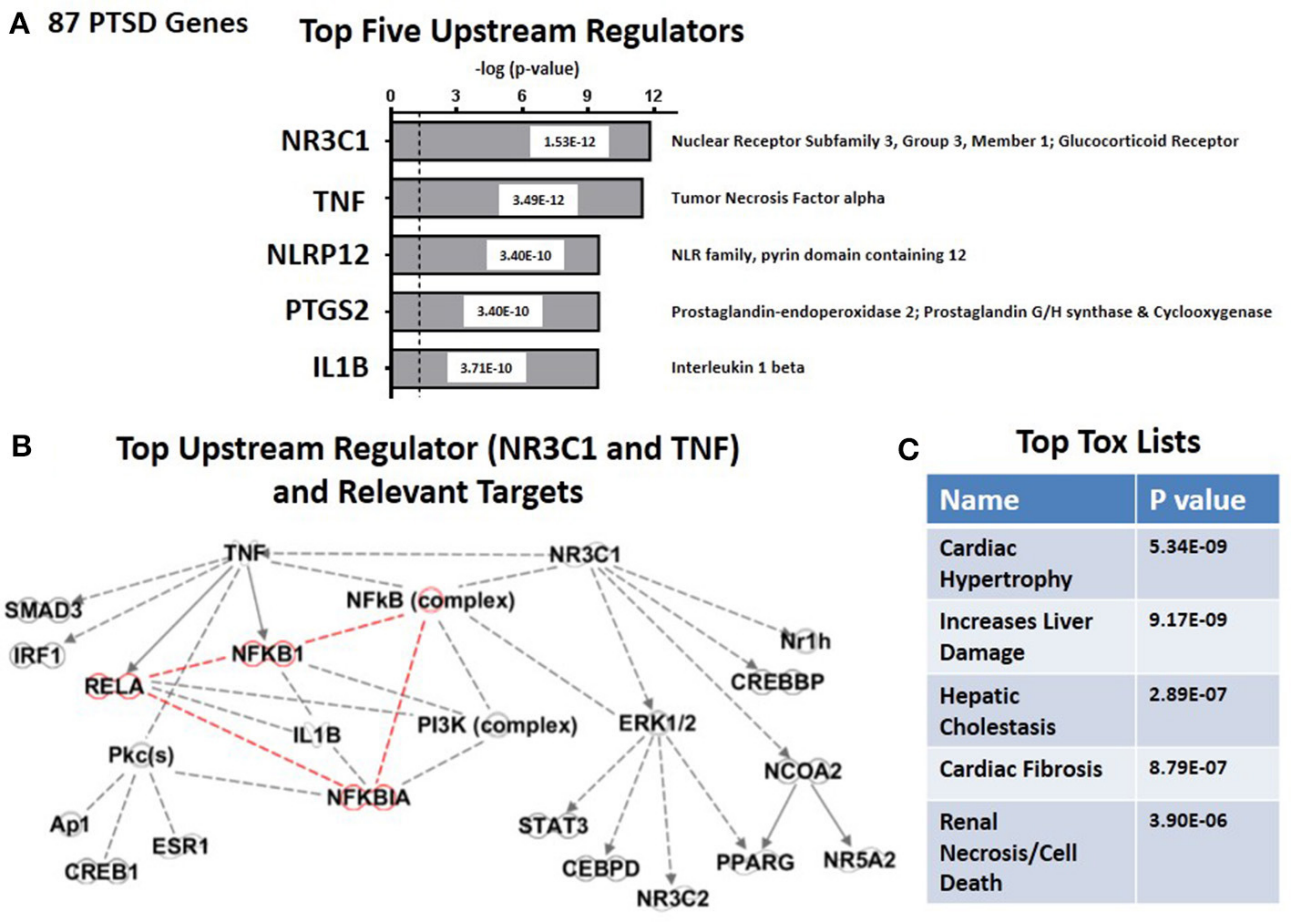
**Upstream regulation of risk genes for PTSD**. **(A)** Predicted Top Five Upstream Regulators. The top two are NR3C1 (Glucocorticoid Receptor) and TNFα. **(B)** Predicted Targets of top two upstream regulator, NR3C1 (Glucocorticoid Receptor) and TNFα. **(C)** Predicted Top Tox (icology) Functions. Top tox function is cardiac hypertrophy.

By using the causal analytics tool “Upstream Regulator Analysis” within the IPA program, we were next able to test whether there might be an upstream integrating mechanism which could act as a “regulator” for PTSD-related risk genes. The advantage of causal network analysis is that it integrates previously observed cause-effect relationships reported in the literature, and is more powerful than gene set enrichment alone since it leverages knowledge about the direction of effects rather than mere associations (Krämer et al., 2014). In addition, if the causal effect of one regulator depends on another in the network, then both regulators should be identified. Figure 2A shows the top five statistically significant upstream regulators for the entire set of 83 PTSD risk genes. These include (i) the glucocorticoid receptor (NR3C1, *P* = 1.5 E-12); (ii) Tumor Necrosis Factor alpha (TNFα, *P* = 3.49E-12), (iii) NLRP12 (*P* = 3.40E-10) PTGS2 (*P* = 3.40 E-10); and (iv) Interleukin 1 beta (IL-1β, *P* = 3.71E-10). What is generally true about these five genes is that they all are related in some way to the process of inflammation. Figure 2B graphs the predicted downstream relationships among the top two regulators: TNFα and the glucocorticoid receptor. What is apparent from this graph is that the two regulators have different downstream consequences, reminiscent to some extent of *Networks #1* and *#2* (see Figures 1B,D). However, the overlap focuses on the “NFκB Complex” (Chen and Greene, 2004), and its activating cytosolic component RELA (NFκB, p65), and its inhibitory cytosolic components NFKB1A (IκBα), and NFKB1(NFκB, p105). These internode interactions are marked in red. It would therefore appear that upstream regulation of the PTSD risk genes might be dominantly regulated by proinflammatory signaling mechanisms. Figure 2C shows that the top predicted toxicology list is headed by cardiac hypertrophy (*P* = 5.34E-09) and liver damage (*P* = 9.17E-09).

### Identification of candidate PTSD-risk genes for which mutations are also associated independently with increased risk for cardiovascular disease

To test the hypothesis that any of the 87 *candidate* risk genes might overlap with risk genes for CVD, we utilized a published set of *candidate* cardiovascular disease (CVD) risk genes [163 genes (Gibbons et al., 2004)]. We also included an additional 309 imputed genes from more recent GWAS data that had assigned risk factors of at least 10% (Cambien and Tiret, 2007; Whitfield, 2014). However, independent validation of GWAS- imputed risk genes has been a challenge (Simon et al., 2016). As shown in Supplemental Table 1 (see “YES” in the CVD column), 36 *candidate* CVD risk genes are common to both PTSD and CVD. Not unexpectedly, none of the GWAS-imputed CVD risk genes overlapped with the *candidate* PTSD risk genes. The association between *candidate* PTSD risk genes and *candidate* CVD risk genes is statistically significant (Fisher's exact test on a 2X2 table [19790, 127;47,36], assuming 20,000 genes in the genome; p = 3 × 10E-54).

**Figure 3:**
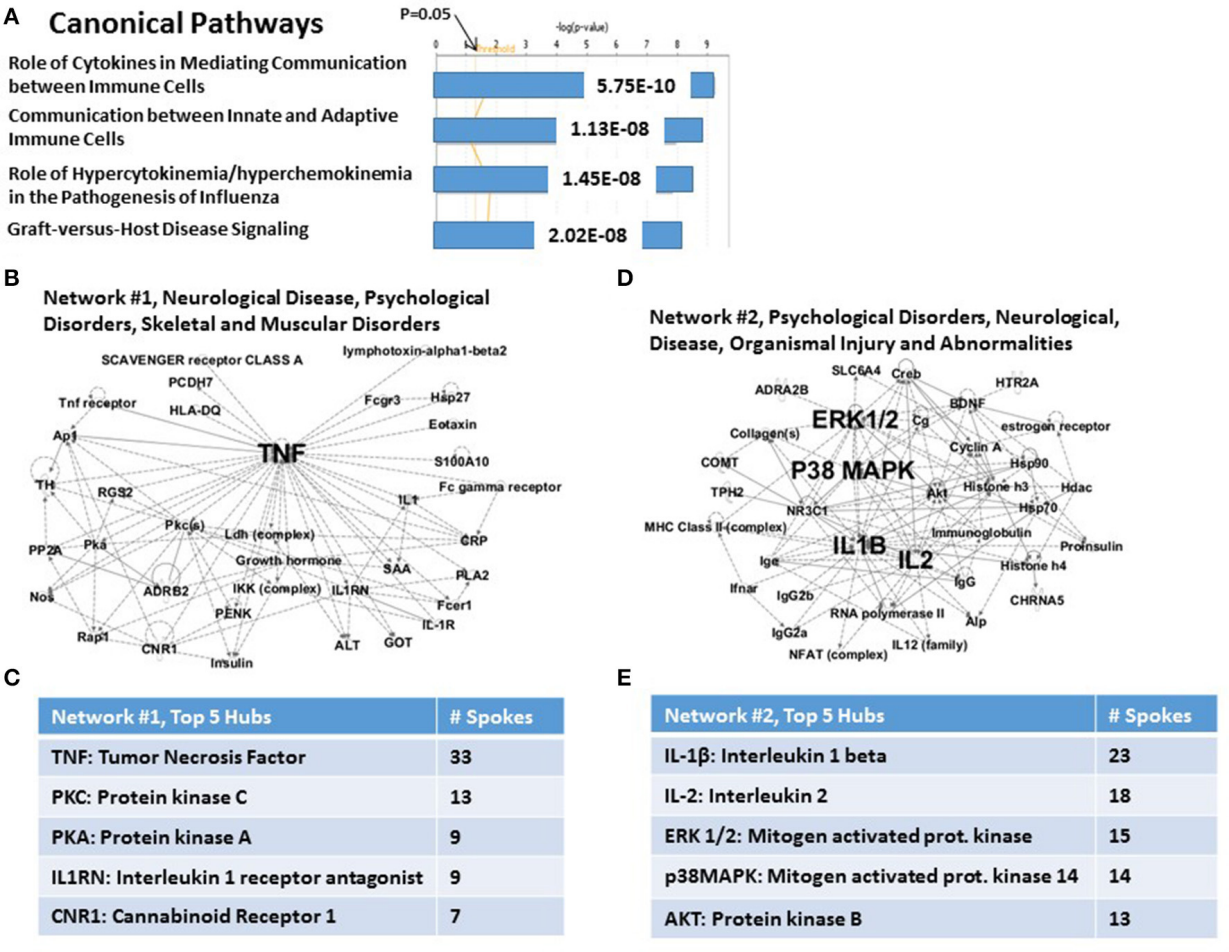
**Functional relationship among 36 candidate PTSD genes, which share an independent risk factor status with cardiovascular disease**. **(A)** Top canonical pathways. The top signaling pathway is Role of Cytokines in Mediating Communication between Immune Cells. **(B)** Associated Network Function #1. The top hub is TNFalpha, **(C)** *Inset*: Network #1, top 5 hubs. **(D)** Associated Network Function #2. The top hubs are IL-1β, IL-2, p38MAPK, and ERK1/2. **(E)** *Inset*: Network #2, top 5 hubs.

A portion of the IPA analysis of this set of 36 CVD genes is shown in Figure 3. Figure 3A shows that the top highly significant Canonical Pathways are dominated by immune functions. These include Role of Cytokines Mediating Communication between Immune Cells (*P* = 5.75E-10); Communication between Innate and Adaptive Immune Cells (*P* = 1.13E-08); Role of Hypercytokinemia/Hyperchemokinemia in the Pathogenesis of Influenza (essentially “cytokine storm,” *P* = 1.45E-08); and Graft-vs.-Host Disease signaling (*P* = 2.02E-08). In support of these signaling pathways are the top two Networks. In the case of *Network #1* (Figure 3B: “Neurological Disease, Psychological Disorders, Skeletal and muscular disorders”), 11 of 33 nodes in this graph are genes from the complete set of 36 CVD risk genes. Figure 3C shows that TNFα is the principal hub which integrates all of the nodes made up of 11 CVD genes and 22 other genes and functions which are inferred to be connected to this set of genes. Other hubs, including PKC, PKA, IL-1RN and CNR1, interact with progressively fewer of the nodes and are presumably progressively less important. For the case of *Network #2* (Figure 3D: “Psychological Disorders, Neurological Disease, Organismal Injury and Abnormalities”), 9 of 31 nodes are genes from the complete set of 36 CVD risk genes. Figure 3E shows that the top two hubs are IL-1β and IL-2, followed by hubs with progressively fewer links: ERK1/2, P38MAPK and AKT/PKB. Network #2 shares no CVD genes with Network #1, and together they therefore provide an integrating mechanism for 20 (*viz*., 56%) of the 36 CVD risk genes. Note that four of the top 5 hubs for the CVD Network #2, are also in the top 5 hubs for the PTSD Network #2 (*viz*., compare Figure 3E with Figure 1E). However, only two genes, IL-2 and BDNF, are nodes in both networks. These relationships suggest that both the complete set of 83 PTSD risk genes, and the sub-set of 36 CVD risk genes, may share at least one genomic signature.

**Figure 4:**
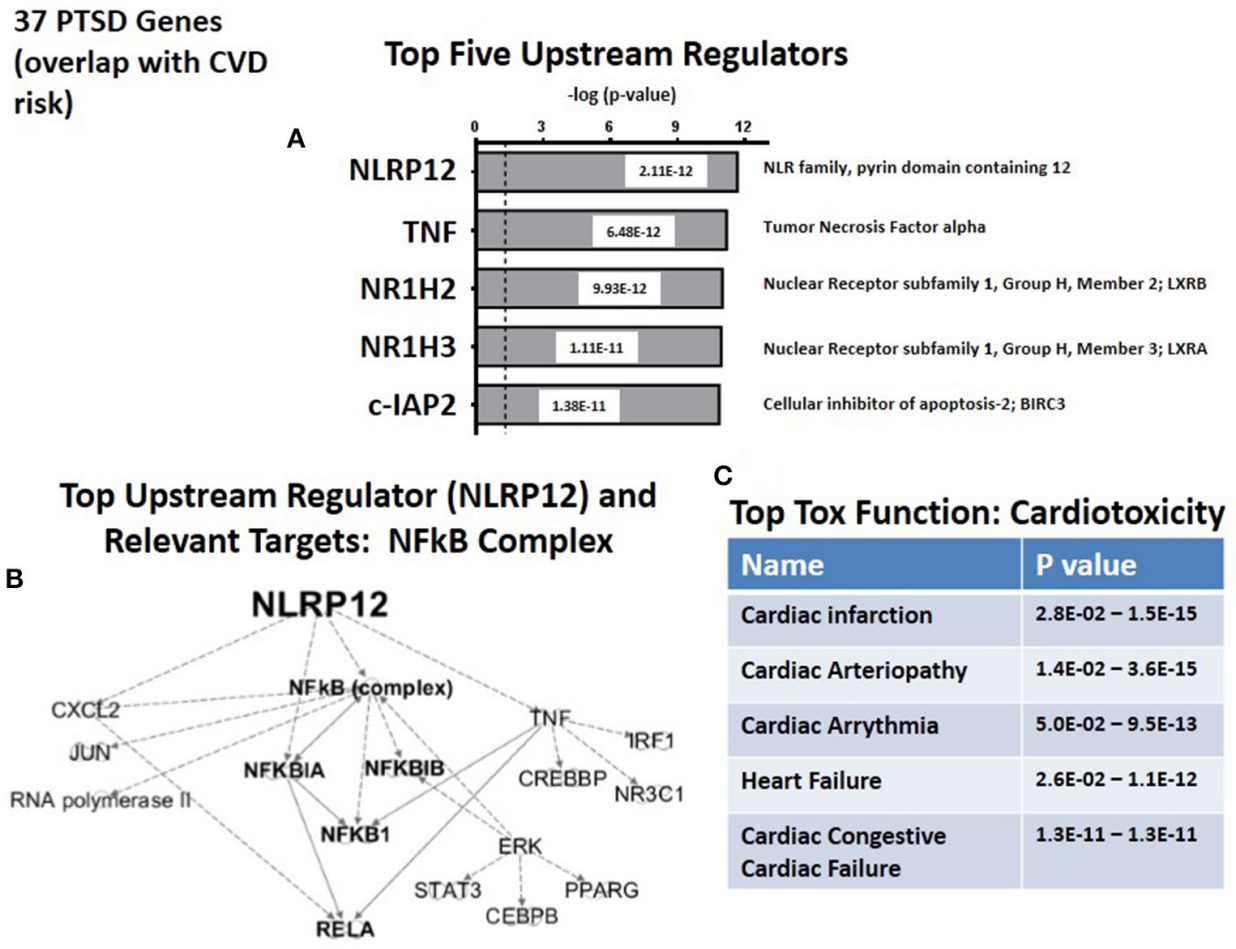
**Upstream regulation of risk genes for CVD. (A)** Predicted Top upstream Regulators. The top two are NLRP12 and TNFa. **(B)** Predicted Targets of top two upstream regulator, NLRP12 and TNFa. **(C)** Predicted Top Tox (icology) Functions. All five describe cardiac disease.

We next used the causal analytics tool “Upstream Regulator Analysis” to test whether there might be an upstream integrating mechanism which could act as a “regulator” for the subset of 36 CVD risk genes. Figure 4A lists the predicted top five upstream CVD regulators, two of which, NLRP12 and TNFα, are the same upstream regulators as those predicted for the complete set of 83 PTSD risk genes (see Figure 2A). Of the remaining 3 regulators, NR1H2 (LXRB) and NR1H3 (LXRA) form a family of heterodimers that control both lipid homeostasis (Schultz et al., 2000) and inflammation (Joseph et al., 2003). Furthermore, recent twin and family GWAS studies have identified LXRA and LXRB genes in signatures for coronary artery disease and hepatic steatosis, and have been interpreted to have broad effects on multiple metabolic traits (Rankinen et al., 2015). The fifth predicted regulator, c-IAP2, is an anti-apoptosis gene which inhibits inflammation-associated TNF receptor signaling and controls downstream NFκB activation (Mayer et al., 2011). c-IAP2 also protects cardiac fibroblasts through suppression of ERK1/2 MAPK and NFκB signaling (Philip and Shivakumar, 2013). Finally, as shown in Figure 4B, the top two regulators, NLRP12 and TNF, identify elements in the NFκB complex as the relevant targets for the Regulators. Therefore, whether working with either the 87 candidate risk genes for PTSD (see Figure 2B), or the sub-set of CVD risk genes (see Figure 4B), the same NFκB complex is strongly identified as a common set of downstream targets. Consistently, Figure 4C identifies cardiotioxicity as the top toxicology function for the CVD risk genes. Included in this function are infarction, arteriopathy, arrhythmia, heart failure, and congestive heart failure. However, given that this sub-set of 36 PTSD genes were all identified as independent risk factors for CDV, this prediction may not be unexpected.

### Identification of candidate PTSD-risk genes for which mutations are also associated independently with increased risk for type 2 diabetes mellitus

As a possible “control” for the association between PTSD and CVD risk genes, we tested for a relationship between candidate PTSD risk genes, and candidate genes for Type 2 Diabetes Mellitus (T2DM). The rationale for this choice was that results from the Viet Nam Twins study had shown a familial relationship between PTSD and CVD (Vaccarino et al., 2013), but failed to show a familial relationship for PTSD and T2DM (Vaccarino et al., 2014). For consistency, we utilized a recently published T2DM risk gene set comprised of 106 *candidate* genes (Kluth et al., 2014). We then calculated the overlap of the T2DM risk gene set and the CVD risk gene set. As shown in Supplementary Table 1, only 15 genes (18%) of the PTSD risk genes were also risk genes for T2DM (see). However, this enrichment was statistically significant (Fisher's exact test on a 2X2 table [19769, 148; 68, 15], assuming 20,000 genes in the genome, *P* = 1.8 × 10^−16^. Furthermore, of these 15 genes, 14 were also in the CVD category.

To further test for the enrichments of PTSD risk genes in the CVD gene set vs. the PTSD enrichment in the T2DM gene set, we compared odds ratios. The odds ratio for PTSD and CVD enrichment was 118.9 (95% CI, [72.4-195.4]). By contrast, the odds ratio for the PTSD and T2DM enrichment was 29.4 (95% CI, [15.26-53.4]). Because the PTSD and CVD odds ratio was greater than the PTSD and T2DM odds ratio, we conclude that PTSD and CVD enrichment is significantly greater than the PTSD and T2DM enrichment. However, it remains important to take into consideration that these odds ratios are not independent.

**Figure 5:**
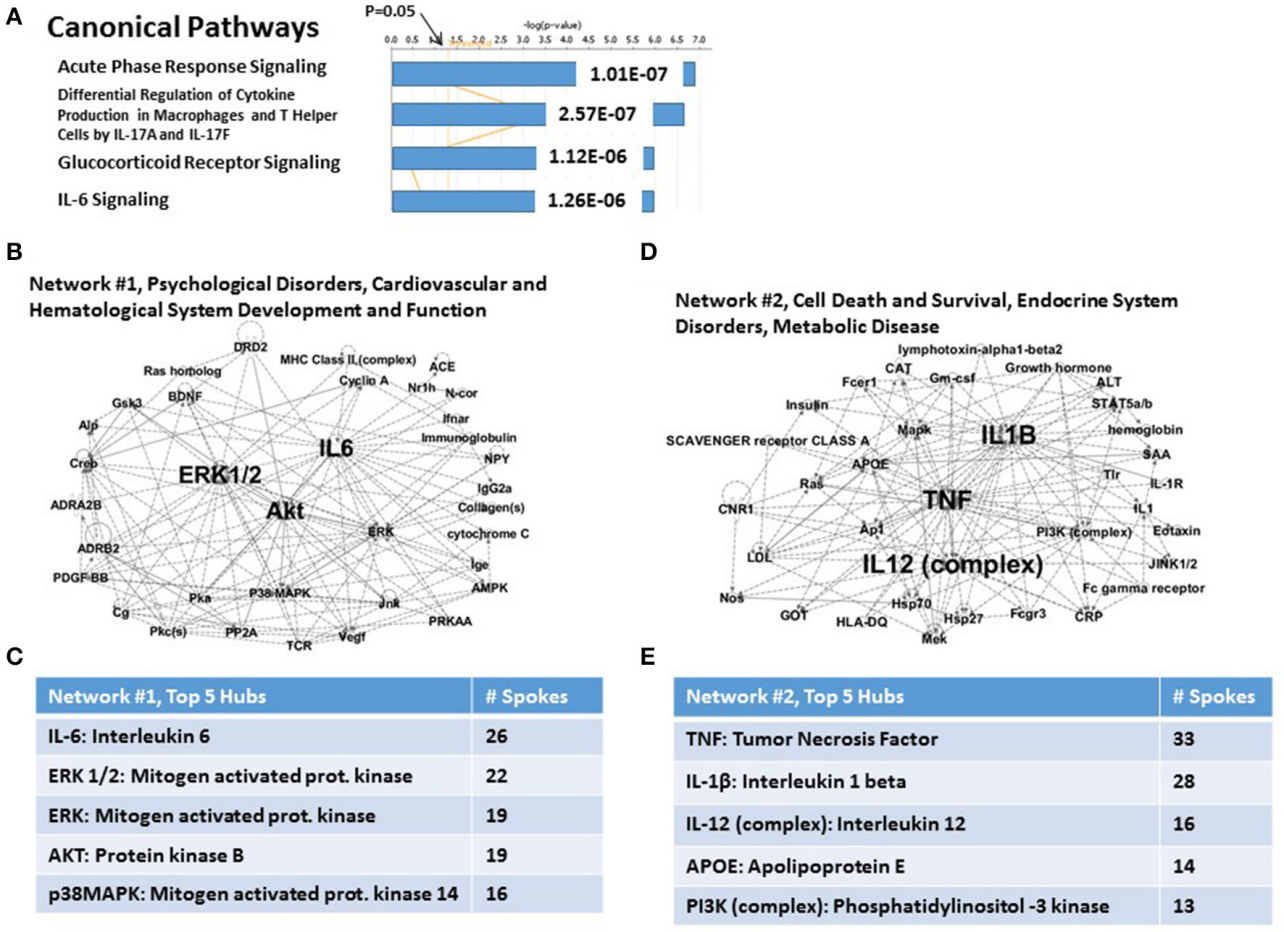
**Functional relationship among 15 candidate T2DM genes, which share an independent risk factor status with PTSD**. **(A)** Top canonical pathways. The top signaling pathway is Acute Phase Response Signaling. **(B)** Associated Network Function #1. The top hubs are IL-6, ERK1/2, ERK, and AKT. **(C)** *Inset*: Network #1, top 5 hubs. **(D)** Associated network Function #2. The top hubs are TNFα, IL-1β and IL-12. **(E)** *Inset*: Network #2, top 5 hubs.

Figure 5A shows that the Canonical Pathways for T2DM risk genes concentrate on immune functions. This was also the case for the set of 34 CVD risk genes; however, for the T2DM risk genes, the specific Canonical Pathways are quite different. Here, these include Acute Phase Response Signaling (*P* = 1.01E-07); Regulation of cytokine production in macrophages and T helper cells by IL-17A and IL-17F (*P* = 2.57E-07); glucocorticoid receptor signaling (*P* = 1.12E-06); and IL-6 signaling (*P* = 1.26E-06). Of potential concern is the fact that the *P*-values for these Canonical pathways are substantially elevated compared to the PTSD risk genes (see Figure 1A) and CVD subset of risk genes (see Figure 3A). However, this finding is very likely dependent on the relatively *reduced number* of total T2DM risk genes available for analysis, and serve as a signpost of the relative paucity of genes in this category, no matter how statistically significant.

The two top Associated Networks for this system are shown in Figures 5B,C, respectively. Network #1 (Figure 5B) is built from only 4 of the 15 T2DM genes, and is marked uniquely by a heavily populated hub for IL-6, and progressively less for 4 mitogen-related genes (see Figure 5C). Network #2 (Figure 5D) is built from 6 of the 15 T2DM genes, and contains heavily populated hubs for TNFa and IL-1β. (see Figure 5E). Progressively fewer linked hubs for Network #2 include the IL-12 complex, apolipoprotein E, and the PI3K complex. Thus, both Associated networks together account for 10 (66.67%) of the 15 T2DM genes. It is also apparent, regardless of significantly different gene numbers, that TNFα and ERK1/2 are major hubs for both CVD risk genes and T2DM risk genes, and that ERK1/2 is also a major hub for one of the network describing the parental set of PTSD risk genes.

**Figure 6:**
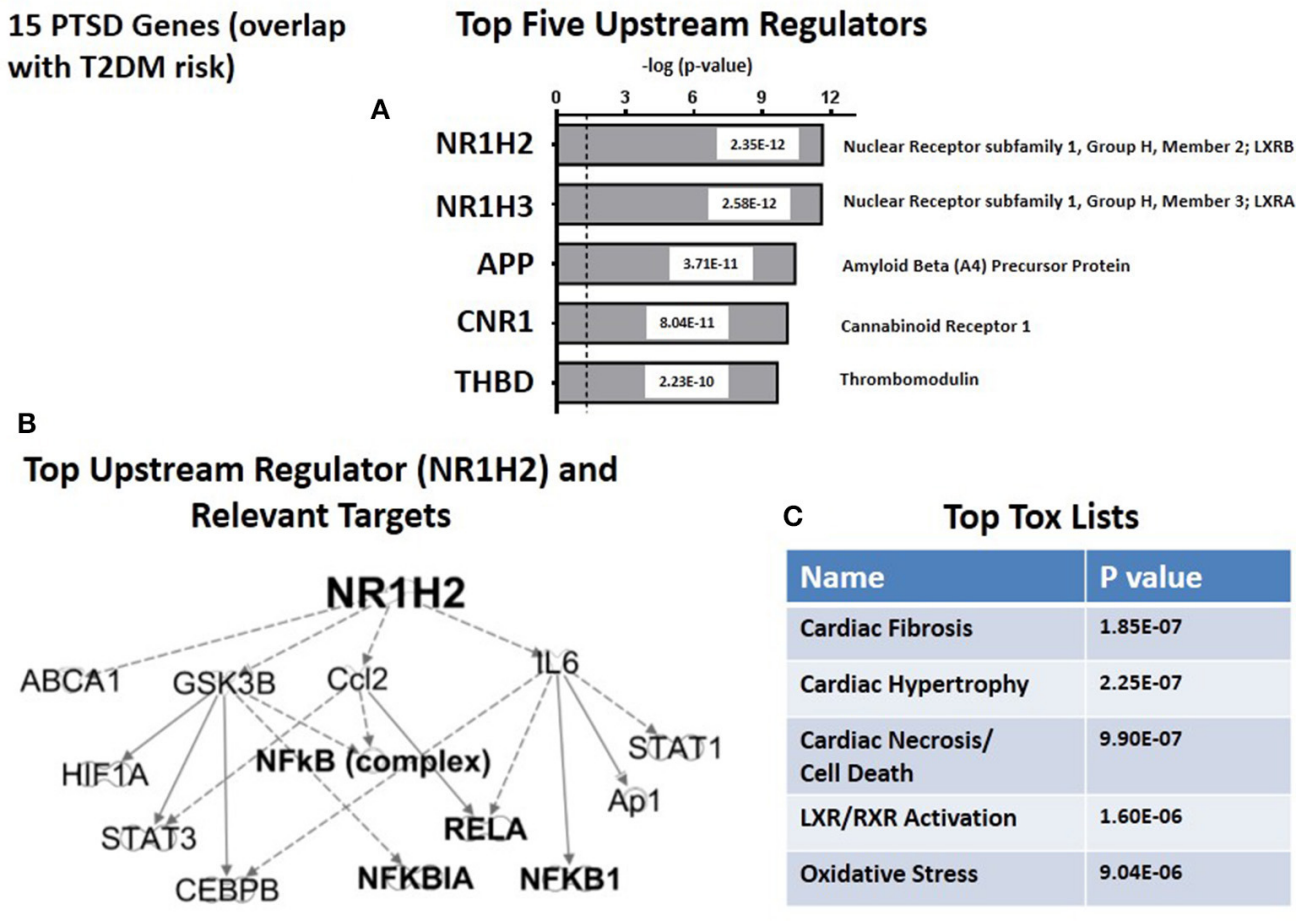
**Upstream regulation of risk genes for T2DM. (A)** Predicted Top Upstream Regulators. The top two are LXRA and LXRB. **(B)** Predicted targets of the top upstream regulator, LXRB. **(C)** Predicted Top Tox (icology) Lists. The emphasisis on cardiac disease.

Figure 6A shows the top five predicted upstream regulators for T2DM risk genes. This list is dominated by the now familiar transcription factors, LXRB and LXRA, and with lesser significance by three other genes: APP (amyloid (A4) Precursor Protein); (CNR1) (cannabinoid Receptor 1) and THBD (thrombomodulin). Consistent with the results from the CVD risk genes (see Figure 5B), the targets for the top upstream regulator, NH1H2/LXRB include the NFκB complex and associated NFκB drivers and inhibitors (see Figure 6B). The top toxicology lists (Figure 6C) focuses on three cardiovascular dysfunctions, and with lower significance both LXR/RXR activation. These address lipid homeostasis and inflammation, and oxidative stress. In retrospect, the fact that 14 of 15 T2DM risk genes are the same as both PTSD and CVD risk genes suggests that the parallel between CVD and T2DM in terms of predicted Canonical Pathways, predicted Associated Networks, and predicted Upstream Regulators should not be unexpected. However, there are quantitative and qualitative differences suggesting that the risk for CVD in PTSD patients may be unique from T2DM risk.

## Discussion

**Figure 7:**
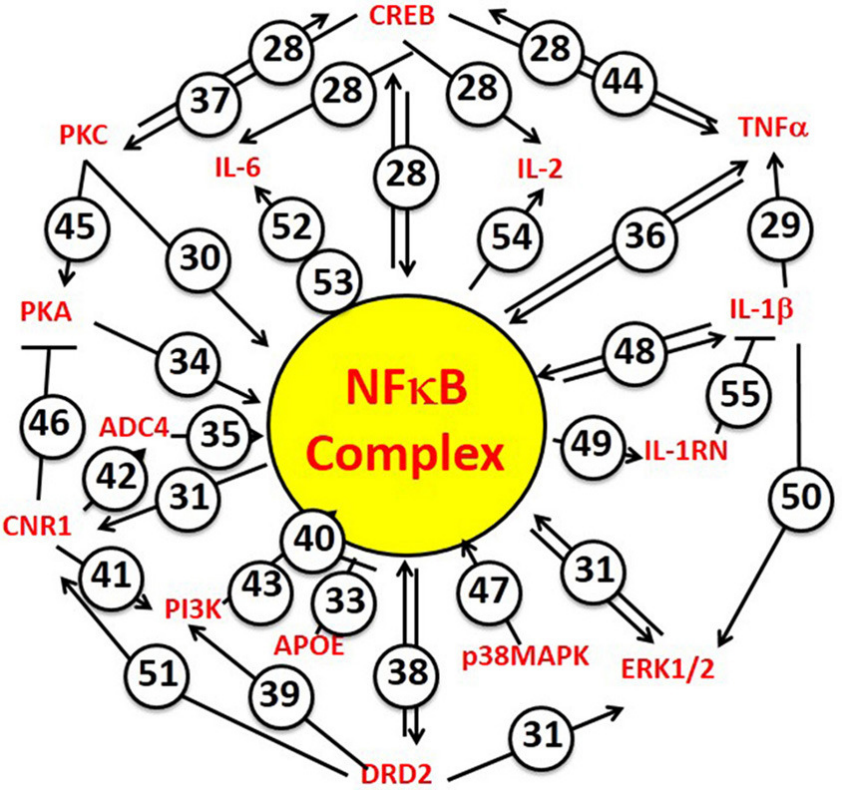
**NFkB Complex is the focus of principal hubs for PTSD and CVD risk genes**. The NFkB Complex is identified specifically by the IPA algorithm to include the cytosolic inhibitors [NFκB, p105] and [IκBα], and the driver [NFκB, p65]. Numbers attached to each arrow are example references from the experimental literature, which document activator functions (arrows) or inhibitor functions (T-endings) between genes. Many of the NFkB-driven genes also interact with each other. When making predictions regarding mechanism, the IPA algorithm takes these interactions into account by reference to its “master” network of information from the Ingenuity Knowledge Base. The numbers correspond to the following references. 28. Wang et al., 2009; Wen et al., 2010; 29. Saperstein et al., 2009; 30. Shirakawa and Mizel, 1989; 31. Chiang et al., 2013; 33. Kim et al., 2013; 34. King et al., 2011; 35. Shirakawa et al., 1989; 36. Zhou et al., 2003; 37. Gonzalez et al., 2015; 38. Takeuchi and Fukunaga, 2004; 39. Nair and Sealfon, 2003; 40. Li et al., 2007; 41. Sánchez et al., 2003; 42. Howlett, 2005; 43. Gustin et al., 2004a; 44. Gustin et al., 2004b; 45. Chen and Roper, 2003; 46. Davis et al., 2003; 47. Wang X. et al., 2012; 48. Wesche et al., 2013; 49. Wang V. Y. et al., 2012; 50. Gao et al., 2006; 51. Patel et al., 2003; 52. Maliner-Stratton et al., 2001; 53. Libermann and Baltimore, 1990; 54. Liao et al., 2013; 55. Liu et al., 2014.

Beginning with the first “soldier's heart” study by Da Costa following the American Civil War, and continuing to the present wars with ever increasing epidemiological sophistication, susceptibility to PTSD following of exposure to intense emotional and physical stress has been found to be accompanied by an increased risk for CVD (Roy et al., 2015). The network analysis of *candidate* gene studies we report here has permitted us to test the hypothesis that the relationship between PTSD and CVD is genetic, and to conclude that the hypothesis has strong and significant support from the available data. (Gill et al., 2009; Gola et al., 2013; Zhou et al., 2014; Breen et al., 2015). Furthermore, the mechanistic driver linking common susceptibility to PTSD and CVD identifies the NFκB complex (*viz*, [NFκB, p105], [NFκB, p65], and [IκBα]). These are the targets of the principal upstream regulators for PTSD and PTSD/CVD risk. These data are thus quite coherent with a lengthy literature, including a recent strain-specific mouse model system (Cho et al., 2014), implicating inflammation as a mechanism for the biological consequences of both post-traumatic stress and cardiovascular disease. Collectively, these results therefore tend to support a *genetic* hypothesis for susceptibility to PTSD and concomitant cardiovascular disease, involving a common NFκB-associated proinflammatory mechanism. In addition, the data also support the concept that there may also be a modest but still significant genetic link between the risk for PTSD and the risk for T2DM. Finally, as a manual complement to the computational focus of the IPA program on NFκB signaling as an integrating upstream mechanism for the PTSD/CVD genetic link, Figure 7 summarizes concrete examples from the literature, which experimentally connect the principal candidate PTSD and CVD risk gene hubs to activation or inhibition of NFκB signaling, and in some cases to each other (Shirakawa and Mizel, 1989; Shirakawa et al., 1989; Libermann and Baltimore, 1990; Maliner-Stratton et al., 2001; Chen and Roper, 2003; Davis et al., 2003; Nair and Sealfon, 2003; Patel et al., 2003; Sánchez et al., 2003; Zhou et al., 2003; Takeuchi and Fukunaga, 2004; Gustin et al., 2004a,b; Howlett, 2005; Gao et al., 2006; Li et al., 2007; Saperstein et al., 2009; Wang et al., 2009; Wen et al., 2010; King et al., 2011; Wang V. Y. et al., 2012; Wang X. et al., 2012; Chiang et al., 2013; Kim et al., 2013; Liao et al., 2013; Wesche et al., 2013; Liu et al., 2014; Gonzalez et al., 2015). So, the analysis does not just depend on a computer search of the literature, but can be validated manually.

However, there are important caveats for data marshaled here in support of the genetic hypothesis for PTSD/CVD susceptibility. Firstly, the PTSD risk gene data are based mostly on *candidate* genes identified from the literature, independent of statistical association. Candidate genes are chosen by investigators based on hypothesis and logical connections to previous studies (Tabor et al., 2002). However, as alluded to above, there was no choice: GWAS studies for PTSD risk genes have been inconsistent with respect to specific loci. For consistency, we used the same approach for both CVD genes and T2DM risk genes. By contrast, GWAS studies have been productive for CVD and T2DM. However, when we extended the CVD and T2DM lists to include imputed genes from respective GWAS studies, no overlaps with the candidate PTSD gene set were found.

A second caveat is the problem of knowing exactly how many genes are actually in the categories of risk for specific chronic diseases. In the case of *candidate* risk genes for PTSD, we used both manual curation and recourse to multiple databases to deduce the specific number. In the case of independent candidate risk genes for CVD, we found at least 163 genes, and used this number for statistical calculations. We conditionally included 309 CVD risk genes deduced from GWAS studies, associated with at least 10% of the CVD risk (Cambien and Tiret, 2007; Whitfield, 2014). In addition, the HUGE and other databases reach out to at least 1.5% CVD risk, identifying approximately 1080 candidate risk genes. For consistency, we wanted to use equivalent *candidate* risk genes for the T2DM comparison. For this purpose we found 106 *candidate* risk genes for Type 2 Diabetes, which had not been specified as to proportional to risk (Whitfield, 2014). Finally, as pointed out recently, the number of significant loci for coronary artery disease and T2DM is proportional to the number of subjects in a GWAS study (Whitfield, 2014). Thus, the GWAS approach alone is intrinsically underpowered with respect to complete identification of disease risk genes.

A third caveat is that both male and female studies have been analyzed together, as have all ethnicities. It may be that females are more susceptible to PTSD than males, as has been frequently reported. However, the largest number of samples from single cohorts have been males from military cohorts. We do not have many females from the same type of military cohort. Rather most of the female cohorts come from such geographically diverse experiences with earthquakes and hurricanes. However, in addition to gender there were also ethnic diversity to consider: Europeans *per se*, European Americans, Africans, African Americans, Pacific Islanders, Asians, Southeast Asians, Hispanics and Native Americans. Faced with such diversity we grouped all patients together as the most stringent test for some common mechanism.

A final caveat is the realization that to comprehensively test the genetic hypothesis for PTSD/CVD susceptibility, it will be necessary focus solely on WGS. In case there was any doubt about the value of WGS relative to the GWAS-based gene imputation method, Wood et al. (2015) found that of 90 low-frequency signals detectable by WGS of 450 individuals, from the InCHIANTI aging study, 57 (i.e., 63%) could not be detected using GWAS imputation alone. Importantly, these authors also determined that single-variant low frequency large effect signals represented only 7% of detectable signals. Whole Genome Sequences from large numbers of well-curated patients and controls is the core strategy in the Precision Medicine paradigm. This paradigm therefore needs to be applied to the problem of identifying the genetic mechanisms, if they exist, which are responsible for common susceptibilities to PTSD, CVD and T2DM (Insel, 2014; Wood et al., 2015).

### Conclusions

Based on a manually curated, computer-assisted, comprehensive analysis of previously published candidate gene studies, we find evidence for a genetic basis for the highly significant parallel susceptiblity to PTSD and CVD by soldiers and civilians exposed to extreme stress. There may also be evidence from this analysis for a genetic linkage between PTSD and T2DM. Our findings further suggest that the fundamental genetic mechanism may involve upstream NFκB-mediated inflammation. However, a precision medicine approach, with larger numbers of well-curated PTSD patients, are needed to comprehensively test both the genetic hypothesis and the candidate genetic mechanism.

## Experimental procedures

### Selection of genes

The data analyzed here for PTSD and CVD were locked in as of May 25, 2015. The data for Type 2 Diabetes mellitus were locked in as of May 26, 2015. For selection of genes, the following databases were searched: PubMed, GWAS catalog from NIH, HuGE navigator and PILOT (Published International Literature On Traumatic Stress) database from the Department of Veterans Affairs. When using PubMed and PILOT database used different keywords (see below): (a) “PTSD”; (b) “Post traumatic stress disorder”; (c) “PTSD and genomics”; (d) “PTSD and SNPs”; (e) “PTSD & Variants”; (f) “Post traumatic stress disorder” and “genomics”; (g)“Post traumatic stress disorder” and “SNPs”; (h)“Post traumatic stress disorder” and “variants”; (i) “PTSD” and “GWAS”' (j) “Post traumatic stress disorder” and “GWAS”. When using the GWAS catalog from NIH, we selected “PTSD” [from the list] for the “disease” section, and used default setting to retrieve the data. In the HuGE navigator, we used the “Pheopedia” and used the disease term “Stress disorders, post-traumatic”. In both cases, we looked at all the publications for significant SNPs on the results/summary or discussion section. We manually read every referenced article to test validity of the claim, and to identify the specific mutation reported. The data for PTSD and the CVD and T2DM subcategories are comprehensively summarized in Supplementary Table 1. By way of a caveat for these “YES” genes, the same concerns regarding power and significance noted for PTSD risk genes also apply to the data for independent CVD risk genes. Nonetheless, candidate gene data have their own coherence and structure, and can explicitly be compared with other data collected in the same manner.

### IPA algorithm

The functional analysis algorithm from Ingenuity Pathway Analysis (IPA; Ingenuity Systems, http://www.ingenuity.com) was used to identify the top canonical pathways, top predicted diseases and disorders associated with these canonical pathways, top physiological systems, and top associated networks. The algorithm transforms a list of genes into a set of relevant networks based on extensive records maintained in the Ingenuity Knowledge Base (IKB). Hub and spoke tables were constructed manually from inspection of the associated networks. Upstream regulators were identified by the Causal Analysis Algorithm, lodged within the parental IPA algorithm, using “master” network information from the Ingenuity Knowledge Base.

### Statistics

The significance of differences in outcomes was computed using Fishers exact test to calculate a two-tailed *P*-value, or McNamar Test for the same purpose, using the STATA program. Significance was taken to be represented by *P* < 0.05.

## Author contributions

HP, CS, OE, DJ, CD, MS, MW, RU, MBS: substantially contributed to the conception and design of the work; revised work critically for important intellectual content; gave final approval of version to be published; agrees to be accountable for all aspects of the work in ensuring all questions related to accuracy and integrity of any part of the work are appropriately investigated and resolved. HP, CS, JS, MW: substantially contributed to acquisition, analysis and interpretation of data for the work; revised work critically for important intellectual content; gave final approval of version to be published; agrees to be accountable for all aspects of the work in ensuring all questions related to accuracy and integrity of any part of the work are appropriately investigated and resolved.

## Funding

This work was funded by the NIH (IAA-A-HL_14-007.001; PI: HP, M.D., Ph.D). CDMRP (W81XWH-08-2-0201; PI: HP, M.D., Ph.D).

### Conflict of interest statement

The authors declare that the research was conducted in the absence of any commercial or financial relationships that could be construed as a potential conflict of interest.
