# Supplementary material for: “Soldier's Heart”: A Genetic Basis for Elevated Cardiovascular Disease Risk Associated with Post-traumatic Stress Disorder
Source: Front Mol Neurosci. 2016 Sep 23;9:87. doi: 10.3389/fnmol.2016.00087 (PMC5033971; doi:10.3389/fnmol.2016.00087)
Supplement: Supplementary file 1 [file Table1.PDF]

	Candidate PTSD Genes	Gene Name	PTSD (association, references)	PTSD (no assoc. ref.	Number of Patients*	CVD Risk Genes	CVD (references)	T2DM Risk genes	T2DM (references)
1	ACE	angiotensin 1 converting enzyme	[1]		3803	YES	[2]	YES	[3]
2	ADCY8	adenylate cyclase 8 (brain)	[4,5]		484				
3	ADCYAP1 R1	adenylate cyclase activating polypeptide 1 (pituitary) receptor type I	[6-9]		2453				
4	ADRA2B	adrenoceptor alpha 2B	[10]		119	YES	[11]	YES	[12]
5	ADRB2	adrenoceptor beta 2, surface	[13]		2893	YES	[14]	YES	[15]
6	ANKK1	ankyrin repeat and kinase domain containing 1	[16]	[17]	63				
7	ANK3	ankyrin 3, node of Ranvier (ankyrin G)	[18]		554				
8	APOE,ε2	apolipoprotein E, ε2	[19]		128	YES	[20]	YES	[21]
9	BDNF	brain-derived neurotrophic factor	[22,23]		150	YES	[24]	YES	[25]
10	CAT	catalase	[26]			YES	[27]	YES	[28]
11	CHRNA5	cholinergic receptor, nicotinic, alpha 5 (neuronal)	[29]		502	YES	[30]		
12	CNR1	cannabinoid receptor 1 (brain)	[31]		187	YES	[32]	YES	[33]
13	COBL	cordon-bleu WH2 repeat protein	[4]		4344				
14	COMT	catechol-O-methyltransferase	[29,34-36]		1777	YES	[37,38]		
15	CRHBP	corticotropin releasing hormone binding protein	[39]		93				
16	CXCL8 (IL-8)	chemokine (C-X-C motif) ligand 8	[40,41]		178	YES	[42,43]		
17	CRHR1	corticotropin releasing hormone receptor 1	[44]		637				
18	CRHR2	corticotropin releasing hormone receptor 2	[45]		491				
19	CRP	C-reactive protein, pentraxin-related	[46]		2692	YES	[47]	YES	[48]
20	DAT	dopamine transporter	[49]		212				
21	DBH	dopamine beta-hydroxylase (dopamine beta-monooxygenase	[50]		167				
22	DDX60L	DEAD (Asp-Glu-Ala-Asp)box polypeptide 60-like	[51]		1708				

23	DPP6	dipeptidyl-peptidase 6	[5]		484	YES	[52]		
24	DRD2	dopamine receptor D2	[16,53,54]	[55]	1749			YES	[56]
25	DRD4	dopamine receptor D4	[57]		107				
26	DTNBP1	dystrobrevin binding protein 1	[58]		377				
27	DUSP2 (PAC1)	dual specificity phosphatase 2	[6]		1200				
28	EDEM1	ER degradation enhancer, mannosidase alpha-like 1	[59]		86				
29	EGR1	early growth response 1	[60]		152				
30	FAAH	fatty acid amide hydrolase	[61]		115	YES	[62]		
31	FKBP5	FK506 binding protein 5	[29,63]		412				
32	GABRA2	gamma-aminobutyric acid (GABA) A receptor, alpha 2	[64]		46				
33	GABRB3	gamma-aminobutyric acid (GABA) A receptor, beta 3	[65]		86				
34	GAD1	glutamate decarboxylase 1 (brain, 67kDa)	[34]		928				
35	HTR1A	5-hydroxytryptamine (serotonin) receptor 1A, G protein-coupled	[66]		297				
36	HTR2A	5-hydroxytryptamine (serotonin) receptor 2A, G protein-coupled	[67]		174	YES	[68]		
37	GRIN2A	glutamate receptor, ionotropic, N-methyl D-aspartate 2A	[54]		1749				
38	GRIN2B	glutamate receptor, ionotropic, N-methyl D-aspartate 2b	[54]		1749				
39	HTR2A	5-hydroxytryptamine (serotonin) receptor 2A, G protein-coupled	[67]		174	YES	[68]		
40	IL1B	interleukin 1, beta	[69,70]		79	YES	[71]	YES	[72]
41	IL1RN	interleukin 1 receptor antagonist	[73]		406	YES	[74]		
42	IL2	interleukin 2	[75]		110	YES	[76]		
43	IL6	interleukin 6	[77]		56	YES	[78]	YES	[79]
44	INPP1	inositol polyphosphate-1-phosphatase	[80]		184	YES	[81]		
45	ITGA6	Integrin alpha 6	[54]		1749				
46	lincRNA AC068718.1	long non-coding RNA Aco6718.1	[82]		423				

47	MT-ATP8	mitochondrially encoded ATP synthase 8	[83]		1238	YES	[84]		
48	MT-ND5	mitochondrially encoded NADH dehydrogenase 5	[83]		1238	YES	[85]		
49	MZB1 (PACAP)	marginal zone B and B1 cell-specific protein	[6]		798				
50	NCAM	neural cell adhesion molecule 1	[54]		1749				
51	NRXN3	Neurexin 3	[54]		1749				
52	NOS1AP	nitric oxide synthase 1 (neuronal) adaptor protein	[86]		358	YES	[87]	YES	[88]
53	NPPB	natriuretic peptide B	[89]		891	YES	[90]		
54	NPY	neuropeptide Y	[91]		502	YES	[92]	YES	[93]
55	NR3C1 (GR)	nuclear receptor subfamily 3, group C, member 1 (Glucocorticoid Receptor)	[94-96]		118	YES	[97]	YES	[98]
56	NTRK2	neurotrophic tyrosine kinase, receptor, type 2	[80]		184				
57	OPRL1	opiate receptor-like 1	[99]		1847				
61	OPRM1	opioid receptor, mu 1	[100]		201				
62	OXTR	oxytocin receptor	[101]		205				
63	PCDH7	protocadherin 7	[51]		1708	YES	[102]		
64	PCLO	piccolo presynaptic cytomatrix protein	[103]		66				
65	PENK	proenkephalin	[104]		4231	YES	[105]		
66	PRKCA	protein kinase C, alpha			394				
67	PRKG1	Protein kinase, cGMP dependent, type 1	[51]		1708	YES	[106]		
68	PRTFDC1	phosphoribosyl transferase domain containing 1	[107]		3494				
69	RGS2	regulator of G-protein signaling 2	[34,101]		1133	YES	[108]		
70	RORA	RAR-related orphan receptor A	[109]	[110]	605				
71	S100A10	S100 calcium binding protein A10	[111]		N/A	YES	[112]		
72	SDC2	syndecan 2	[51]		949				
73	SLC18A2	solute carrier family 18 (vesicular monoamine transporter), member 2	[104]		2538	YES	[113]		
74	SLC1A1	solute carrier family 1 (neuronal/epithelial high affinity glutamate transporter, system Xag), member 1	[23,114]		818				

75	SLC6A3	solute carrier family 6 (neurotransmitter transporter), member 3	[23]		2591				
76	SLC6A4	solute carrier family 6 (neurotransmitter transporter), member 4	[34,115,116]		2707	YES	[117]		
77	SRD5A2	steroid-5-alpha-reductase, alpha polypeptide 2 (3-oxo-5 alpha-steroid delta 4-dehydrogenase alpha 2)	[118]		1443				
78	STMN1	stathmin 1	[119]		326				
79	TBC1D2	TBC1 domain family, member 2	[51]		1708				
80	TH	tyrosine hydroxylase	[54]		1749	YES	[120]		
81	TLL1	tolloid-like 1	[4]		1578	YES	[121]		
82	TNF-alpha	tumor necrosis factor alpha	[69]		35	YES	[122-124]	YES	[125]
83	TPH1	tryptophan hydroxylase 1	[126]		200				
84	TPH2	tryptophan hydroxylase 2	[35,126]		400	YES	[127]		
85	TRPS1	trichorhinalphalangeal syndrome 1	[4]		1578				
86	UNCT3C	Unc-13 homolog C (C.elegans)	[51]		1708				
87	WWC1	WW and C2 domain containing 1	[128]		791				

* Patients: number of PTSD and Healthy Control patients in the specific study or studies.

References for Table 1

1. Nylocks KM, Michopoulos V, Rothbaum AO, Almli L, Gillespie CF, et al. (2015) An angiotensin-converting enzyme (ACE) polymorphism may mitigate the effects of angiotensin-pathway medications on posttraumatic stress symptoms. Am J Med Genet B Neuropsychiatr Genet 168b: 307-315.
2. Alazhary NM, Morsy MM, Al-Harbi KM (2015) Angiotensin-converting enzyme gene insertion deletion (ACE I/D) polymorphism in Saudi children with congenital heart disease. Eur Rev Med Pharmacol Sci 19: 2026-2030.
3. Chhabra KH, Chodavarapu H, Lazartigues E (2013) Angiotensin converting enzyme 2: a new important player in the regulation of glycemia. IUBMB Life 65: 731-738.
4. Xie P, Kranzler HR, Yang C, Zhao H, Farrer LA, et al. (2013) Genome-wide association study identifies new susceptibility loci for posttraumatic stress disorder. Biol Psychiatry 74: 656-663.
5. Wolf EJ, Rasmusson AM, Mitchell KS, Logue MW, Baldwin CT, et al. (2014) A genome-wide association study of clinical symptoms of dissociation in a trauma-exposed sample. Depress Anxiety 31: 352-360.
6. Ressler KJ, Mercer KB, Bradley B, Jovanovic T, Mahan A, et al. (2011) Post-traumatic stress disorder is associated with PACAP and the PAC1 receptor. Nature 470: 492-497.
7. Almli LM, Mercer KB, Kerley K, Feng H, Bradley B, et al. (2013) ADCYAP1R1 genotype associates with post-traumatic stress symptoms in highly traumatized African-American females. Am J Med Genet B Neuropsychiatr Genet 162b: 262-272.
8. Uddin M, Chang SC, Zhang C, Ressler K, Mercer KB, et al. (2013) Adcyap1r1 genotype, posttraumatic stress disorder, and depression among women exposed to childhood maltreatment. Depress Anxiety 30: 251-258.
9. Wang L, Cao C, Wang R, Qing Y, Zhang J, et al. (2013) PAC1 receptor (ADCYAP1R1) genotype is associated with PTSD's emotional numbing symptoms in Chinese earthquake survivors. J Affect Disord 150: 156-159.
10. Gibbs AA, Bautista CE, Mowlem FD, Naudts KH, Duka T (2013) Alpha 2B adrenoceptor genotype moderates effect of reboxetine on negative emotional memory bias in healthy volunteers. J Neurosci 33: 17023-17028.
11. Oh SH, Min KT, Jeon YJ, Kim MH, Kim OJ, et al. (2013) Association between common genetic variants of alpha2A-, alpha2B-, and alpha2C-adrenergic receptors and ischemic stroke. Clin Neurol Neurosurg 115: 26-31.
12. Chen QJ, Lu L, Jin C, Wang LJ, Zhang RY, et al. (2010) Insertion/insertion genotype of alpha(2B)-adrenergic receptor gene polymorphism is associated with silent myocardial ischemia in patients with type 2 diabetes mellitus. Clin Biochem 43: 1201-1204.
13. Liberzon I, King AP, Ressler KJ, Almli LM, Zhang P, et al. (2014) Interaction of the ADRB2 gene polymorphism with childhood trauma in predicting adult symptoms of posttraumatic stress disorder. JAMA Psychiatry 71: 1174-1182.
14. Petersen M, Andersen JT, Jimenez-Solem E, Broedbaek K, Afzal S, et al. (2012) Effect of specific ADRB1/ADRB2/AGT genotype combinations on the association between survival and carvedilol treatment in chronic heart failure: a substudy of the ECHOS trial. Pharmacogenet Genomics 22: 709-715.
15. Kilpelainen TO, Lakka TA, Laaksonen DE, Mager U, Salopuro T, et al. (2008) Interaction of single nucleotide polymorphisms in ADRB2, ADRB3, TNF, IL6, IGF1R, LIPC, LEPR, and GHRL with physical activity on the risk of type 2 diabetes mellitus and changes in characteristics of the metabolic syndrome: The Finnish Diabetes Prevention Study. Metabolism 57: 428-436.

16. Huertas E, Ponce G, Koeneke MA, Poch C, Espana-Serrano L, et al. (2010) The D2 dopamine receptor gene variant C957T affects human fear conditioning and aversive priming. Genes Brain Behav 9: 103-109.
17. White MJ, Morris CP, Lawford BR, Young RM (2008) Behavioral phenotypes of impulsivity related to the ANKK1 gene are independent of an acute stressor. Behav Brain Funct 4: 54.
18. Logue MW, Solovieff N, Leussis MP, Wolf EJ, Melista E, et al. (2013) The ankyrin-3 gene is associated with posttraumatic stress disorder and externalizing comorbidity. Psychoneuroendocrinology 38: 2249-2257.
19. Kim TY, Chung HG, Shin HS, Kim SJ, Choi JH, et al. (2013) Apolipoprotein E gene polymorphism, alcohol use, and their interactions in combat-related posttraumatic stress disorder. Depress Anxiety 30: 1194-1201.
20. Tiret L, de Knijff P, Menzel HJ, Ehnholm C, Nicaud V, et al. (1994) ApoE polymorphism and predisposition to coronary heart disease in youths of different European populations. The EARS Study. European Atherosclerosis Research Study. Arterioscler Thromb 14: 1617-1624.
21. Alharbi KK, Khan IA, Syed R (2014) Association of apolipoprotein E polymorphism with type 2 diabetes mellitus in a Saudi population. DNA Cell Biol 33: 637-641.
22. Hemmings SM, Martin LI, Klopper M, van der Merwe L, Aitken L, et al. (2013) BDNF Val66Met and DRD2 Taq1A polymorphisms interact to influence PTSD symptom severity: a preliminary investigation in a South African population. Prog Neuropsychopharmacol Biol Psychiatry 40: 273-280.
23. Valente NL, Vallada H, Cordeiro Q, Miguita K, Bressan RA, et al. (2011) Candidate-gene approach in posttraumatic stress disorder after urban violence: association analysis of the genes encoding serotonin transporter, dopamine transporter, and BDNF. J Mol Neurosci 44: 59-67.
24. Fukushima A, Kinugawa S, Homma T, Masaki Y, Furihata T, et al. (2015) Serum Brain-Derived Neurotrophic Factor Level Predicts Adverse Clinical Outcomes in Patients with Heart Failure. J Card Fail.
25. Xi B, Takeuchi F, Meirhaeghe A, Kato N, Chambers JC, et al. (2014) Associations of genetic variants in/near body mass index-associated genes with type 2 diabetes: a systematic meta-analysis. Clin Endocrinol (Oxf) 81: 702-710.
26. Duan ZX, Li W, Kang JY, Zhang JY, Chen KJ, et al. (2014) Clinical relevance of tag single nucleotide polymorphisms within the CAT gene in patients with PTSD in the Chongqing Han population. Int J Clin Exp Pathol 7: 1724-1732.
27. Nivet-Antoine V, Labat C, El Shamieh S, Dulcire X, Cottart CH, et al. (2013) Relationship between catalase haplotype and arterial aging. Atherosclerosis 227: 100-105.
28. Goth L, Nagy T, Kosa Z, Fejes Z, Bhattoa HP, et al. (2012) Effects of rs769217 and rs1001179 polymorphisms of catalase gene on blood catalase, carbohydrate and lipid biomarkers in diabetes mellitus. Free Radic Res 46: 1249-1257.
29. Boscarino JA, Erlich PM, Hoffman SN, Zhang X (2012) Higher FKBP5, COMT, CHRNA5, and CRHR1 allele burdens are associated with PTSD and interact with trauma exposure: implications for neuropsychiatric research and treatment. Neuropsychiatr Dis Treat 8: 131-139.
30. Zhu Y, Yang J, Yeh F, Cole SA, Haack K, et al. (2014) Joint association of nicotinic acetylcholine receptor variants with abdominal obesity in American Indians: the Strong Heart Family Study. PLoS One 9: e102220.
31. Lu AT, Ogdie MN, Jarvelin MR, Moilanen IK, Loo SK, et al. (2008) Association of the cannabinoid receptor gene (CNR1) with ADHD and post-traumatic stress disorder. Am J Med Genet B Neuropsychiatr Genet 147b: 1488-1494.

32. Liu R, Zhang Y (2011) G1359A polymorphism in the cannabinoid receptor-1 gene is associated with coronary artery disease in the Chinese Han population. Clin Lab 57: 689-693.
33. Buraczynska M, Wacinski P, Zukowski P, Dragan M, Ksiazek A (2014) Common polymorphism in the cannabinoid type 1 receptor gene (CNR1) is associated with microvascular complications in type 2 diabetes. J Diabetes Complications 28: 35-39.
34. Hettema JM, Chen X, Sun C, Brown TA (2015) Direct, indirect and pleiotropic effects of candidate genes on internalizing disorder psychopathology. Psychol Med: 1-10.
35. Goenjian AK, Noble EP, Steinberg AM, Walling DP, Stepanyan ST, et al. (2014) Association of COMT and TPH-2 genes with DSM-5 based PTSD symptoms. J Affect Disord 172c: 472-478.
36. Clark R, DeYoung CG, Sponheim SR, Bender TL, Polusny MA, et al. (2013) Predicting post-traumatic stress disorder in veterans: interaction of traumatic load with COMT gene variation. J Psychiatr Res 47: 1849-1856.
37. Hintsanen M, Elovainio M, Puttonen S, Kivimaki M, Lehtimaki T, et al. (2008) Val/Met polymorphism of the COMT gene moderates the association between job strain and early atherosclerosis in young men. J Occup Environ Med 50: 649-657.
38. Htun NC, Miyaki K, Song Y, Ikeda S, Shimbo T, et al. (2011) Association of the catechol-O-methyl transferase gene Val158Met polymorphism with blood pressure and prevalence of hypertension: interaction with dietary energy intake. Am J Hypertens 24: 1022-1026.
39. Davydow DS, Kohen R, Hough CL, Tracy JH, Zatzick D, et al. (2014) A pilot investigation of the association of genetic polymorphisms regulating corticotrophin-releasing hormone with posttraumatic stress and depressive symptoms in medical-surgical intensive care unit survivors. J Crit Care 29: 101-106.
40. Guo M, Liu T, Guo JC, Jiang XL, Chen F, et al. (2012) Study on serum cytokine levels in posttraumatic stress disorder patients. Asian Pac J Trop Med 5: 323-325.
41. Song Y, Zhou D, Guan Z, Wang X (2007) Disturbance of serum interleukin-2 and interleukin-8 levels in posttraumatic and non-posttraumatic stress disorder earthquake survivors in northern China. Neuroimmunomodulation 14: 248-254.
42. Martynowicz H, Janus A, Nowacki D, Mazur G (2014) The role of chemokines in hypertension. Adv Clin Exp Med 23: 319-325.
43. Apostolakis S, Vogiatzi K, Amanatidou V, Spandidos DA (2009) Interleukin 8 and cardiovascular disease. Cardiovasc Res 84: 353-360.
44. Boscarino JA (2011) Post-traumatic stress disorder and cardiovascular disease link: time to identify specific pathways and interventions. Am J Cardiol 108: 1052-1053.
45. Wolf EJ, Mitchell KS, Logue MW, Baldwin CT, Reardon AF, et al. (2013) Corticotropin releasing hormone receptor 2 (CRHR-2) gene is associated with decreased risk and severity of posttraumatic stress disorder in women. Depress Anxiety 30: 1161-1169.
46. Michopoulos V, Rothbaum AO, Jovanovic T, Almli LM, Bradley B, et al. (2015) Association of CRP genetic variation and CRP level with elevated PTSD symptoms and physiological responses in a civilian population with high levels of trauma. Am J Psychiatry 172: 353-362.
47. Shen J, Ordovas JM (2009) Impact of genetic and environmental factors on hsCRP concentrations and response to therapeutic agents. Clin Chem 55: 256-264.

48. Curocichin G, Wu Y, McDade TW, Kuzawa CW, Borja JB, et al. (2011) Single-nucleotide polymorphisms at five loci are associated with C-reactive protein levels in a cohort of Filipino young adults. J Hum Genet 56: 823-827.
49. Bailey JN, Goenjian AK, Noble EP, Walling DP, Ritchie T, et al. (2010) PTSD and dopaminergic genes, DRD2 and DAT, in multigenerational families exposed to the Spitak earthquake. Psychiatry Res 178: 507-510.
50. Mustapic M, Pivac N, Kozaric-Kovacic D, Dezeljin M, Cubells JF, et al. (2007) Dopamine beta-hydroxylase (DBH) activity and -1021C/T polymorphism of DBH gene in combat-related post-traumatic stress disorder. Am J Med Genet B Neuropsychiatr Genet 144b: 1087-1089.
51. Ashley-Koch AE, Garrett ME, Gibson J, Liu Y, Dennis MF, et al. (2015) Genome-wide association study of posttraumatic stress disorder in a cohort of Iraq-Afghanistan era veterans. J Affect Disord 184: 225-234.
52. Alders M, Koopmann TT, Christiaans I, Postema PG, Beekman L, et al. (2009) Haplotype-sharing analysis implicates chromosome 7q36 harboring DPP6 in familial idiopathic ventricular fibrillation. Am J Hum Genet 84: 468-476.
53. Comings DE, Comings BG, Muhleman D, Dietz G, Shahbahrami B, et al. (1991) The dopamine D2 receptor locus as a modifying gene in neuropsychiatric disorders. Jama 266: 1793-1800.
54. Nelson EC, Heath AC, Lynskey MT, Agrawal A, Henders AK, et al. (2014) PTSD risk associated with a functional DRD2 polymorphism in heroin-dependent cases and controls is limited to amphetamine-dependent individuals. Addict Biol 19: 700-707.
55. Gelernter J, Southwick S, Goodson S, Morgan A, Nagy L, et al. (1999) No association between D2 dopamine receptor (DRD2) "A" system alleles, or DRD2 haplotypes, and posttraumatic stress disorder. Biol Psychiatry 45: 620-625.
56. Guigas B, de Leeuw van Weenen JE, van Leeuwen N, Simonis-Bik AM, van Haeften TW, et al. (2014) Sex-specific effects of naturally occurring variants in the dopamine receptor D2 locus on insulin secretion and type 2 diabetes susceptibility. Diabet Med 31: 1001-1008.
57. Dragan WL, Oniszczenko W (2009) The association between dopamine D4 receptor exon III polymorphism and intensity of PTSD symptoms among flood survivors. Anxiety Stress Coping 22: 483-495.
58. Voisey J, Swagell CD, Hughes IP, Connor JP, Lawford BR, et al. (2010) A polymorphism in the dysbindin gene (DTNBP1) associated with multiple psychiatric disorders including schizophrenia. Behav Brain Funct 6: 41.
59. Nevell L, Zhang K, Aiello AE, Koenen K, Galea S, et al. (2014) Elevated systemic expression of ER stress related genes is associated with stress-related mental disorders in the Detroit Neighborhood Health Study. Psychoneuroendocrinology 43: 62-70.
60. Vukojevic V, Kolassa IT, Fastenrath M, Gschwind L, Spalek K, et al. (2014) Epigenetic modification of the glucocorticoid receptor gene is linked to traumatic memory and post-traumatic stress disorder risk in genocide survivors. 34: 10274-10284.
61. Pardini M, Krueger F, Koenigs M, Raymont V, Hodgkinson C, et al. (2012) Fatty-acid amide hydrolase polymorphisms and post-traumatic stress disorder after penetrating brain injury. Transl Psychiatry 2: e75.
62. Mukhopadhyay P, Horvath B, Rajesh M, Matsumoto S, Saito K, et al. (2011) Fatty acid amide hydrolase is a key regulator of endocannabinoid-induced myocardial tissue injury. Free Radic Biol Med 50: 179-195.
63. Zannas AS, Binder EB (2014) Gene-environment interactions at the FKBP5 locus: sensitive periods, mechanisms and pleiotropism. Genes Brain Behav 13: 25-37.

64. Nelson EC, Agrawal A, Pergadia ML, Lynskey MT, Todorov AA, et al. (2009) Association of childhood trauma exposure and GABRA2 polymorphisms with risk of posttraumatic stress disorder in adults. Mol Psychiatry 14: 234-235.
65. Feusner J, Ritchie T, Lawford B, Young RM, Kann B, et al. (2001) GABA(A) receptor beta 3 subunit gene and psychiatric morbidity in a post-traumatic stress disorder population. Psychiatry Res 104: 109-117.
66. Lee HJ, Lee MS, Kang RH, Kim H, Kim SD, et al. (2005) Influence of the serotonin transporter promoter gene polymorphism on susceptibility to posttraumatic stress disorder. Depress Anxiety 21: 135-139.
67. Kim KH, Woo HY, Lim SW (2008) Association Study of a Serotonin Receptor 2A Gene -1438A/G Polymorphism and Anxiety-Related Traits. Psychiatry Investig 5: 244-246.
68. Fujita M, Minamino T, Sanada S, Asanuma H, Hirata A, et al. (2004) Selective blockade of serotonin 5-HT2A receptor increases coronary blood flow via augmented cardiac nitric oxide release through 5-HT1B receptor in hypoperfused canine hearts. J Mol Cell Cardiol 37: 1219-1223.
69. Gola H, Engler H, Sommershof A, Adenauer H, Kolassa S, et al. (2013) Posttraumatic stress disorder is associated with an enhanced spontaneous production of pro-inflammatory cytokines by peripheral blood mononuclear cells. BMC Psychiatry 13: 40.
70. Spivak B, Shohat B, Mester R, Avraham S, Gil-Ad I, et al. (1997) Elevated levels of serum interleukin-1 beta in combat-related posttraumatic stress disorder. Biol Psychiatry 42: 345-348.
71. Spears LD, Razani B, Semenkovich CF (2013) Interleukins and atherosclerosis: a dysfunctional family grows. Cell Metab 18: 614-616.
72. Stefanidis I, Kreuer K, Dardiotis E, Arampatzis S, Eleftheriadis T, et al. (2014) Association between the interleukin-1beta Gene (IL1B) C-511T polymorphism and the risk of diabetic nephropathy in type 2 diabetes: a candidate-gene association study. DNA Cell Biol 33: 463-468.
73. Loncar Z, Curic G, Mestrovic AH, Mickovic V, Bilic M (2013) Do IL-1B and IL-1RN modulate chronic low back pain in patients with post-traumatic stress disorder? Coll Antropol 37: 1237-1244.
74. (2015) Cardiometabolic effects of genetic upregulation of the interleukin 1 receptor antagonist: a Mendelian randomisation analysis. Lancet Diabetes Endocrinol 3: 243-253.
75. Smith AK, Conneely KN, Kilaru V, Mercer KB, Weiss TE, et al. (2011) Differential immune system DNA methylation and cytokine regulation in post-traumatic stress disorder. Am J Med Genet B Neuropsychiatr Genet 156b: 700-708.
76. Ding R, Gao W, Ostrodci DH, He Z, Song Y, et al. (2013) Effect of interleukin-2 level and genetic variants on coronary artery disease. Inflammation 36: 1225-1231.
77. Pervanidou P, Kolaitis G, Charitaki S, Margeli A, Ferentinos S, et al. (2007) Elevated morning serum interleukin (IL)-6 or evening salivary cortisol concentrations predict posttraumatic stress disorder in children and adolescents six months after a motor vehicle accident. Psychoneuroendocrinology 32: 991-999.
78. Bazina A, Sertic J, Mismas A, Lovric T, Poljakovic Z, et al. (2015) PPARgamma and IL-6 - 174G>C gene variants in Croatian patients with ischemic stroke. Gene 560: 200-204.
79. Nadeem A, Mumtaz S, Naveed AK, Aslam M, Siddiqui A, et al. (2015) Gene-gene, gene-environment, gene-nutrient interactions and single nucleotide polymorphisms of inflammatory cytokines. World J Diabetes 6: 642-647.

80. Bremer T, Diamond C, McKinney R, Shehktman T, Barrett TB, et al. (2007) The pharmacogenetics of lithium response depends upon clinical co-morbidity. Mol Diagn Ther 11: 161-170.
81. Woodcock EA, Wang BH, Arthur JF, Lennard A, Matkovich SJ, et al. (2002) Inositol polyphosphate 1-phosphatase is a novel antihypertrophic factor. J Biol Chem 277: 22734-22742.
82. Guffanti G, Galea S, Yan L, Roberts AL, Solovieff N, et al. (2013) Genome-wide association study implicates a novel RNA gene, the lincRNA AC068718.1, as a risk factor for post-traumatic stress disorder in women. Psychoneuroendocrinology 38: 3029-3038.
83. Flaquer A, Baumbach C, Ladwig KH, Kriebel J, Waldenberger M, et al. (2015) Mitochondrial genetic variants identified to be associated with posttraumatic stress disorder. Transl Psychiatry 5: e524.
84. Matam K, Shaik NA, Aggarwal S, Diwale S, Banaganapalli B, et al. (2014) Evidence for the presence of somatic mitochondrial DNA mutations in right atrial appendage tissues of coronary artery disease patients. Mol Genet Genomics 289: 533-540.
85. Sobenin IA, Sazonova MA, Postnov AY, Bobryshev YV, Orekhov AN (2012) Mitochondrial mutations are associated with atherosclerotic lesions in the human aorta. Clin Dev Immunol 2012: 832464.
86. Lawford BR, Morris CP, Swagell CD, Hughes IP, Young RM, et al. (2013) NOS1AP is associated with increased severity of PTSD and depression in untreated combat veterans. J Affect Disord 147: 87-93.
87. Liu X, Pei J, Hou C, Liu N, Chu J, et al. (2014) A common NOS1AP genetic polymorphism, rs12567209 G>A, is associated with sudden cardiac death in patients with chronic heart failure in the Chinese Han population. J Card Fail 20: 244-251.
88. Chu AY, Coresh J, Arking DE, Pankow JS, Tomaselli GF, et al. (2010) NOS1AP variant associated with incidence of type 2 diabetes in calcium channel blocker users in the Atherosclerosis Risk in Communities (ARIC) study. Diabetologia 53: 510-516.
89. Xue Y, Taub PR, Iqbal N, Fard A, Wentworth B, et al. (2012) Cardiac biomarkers, mortality, and post-traumatic stress disorder in military veterans. Am J Cardiol 109: 1215-1218.
90. Maisel A (2001) B-type natriuretic peptide levels: diagnostic and therapeutic potential. Cardiovasc Toxicol 1: 159-164.
91. Lappalainen J, Kranzler HR, Malison R, Price LH, Van Dyck C, et al. (2002) A functional neuropeptide Y Leu7Pro polymorphism associated with alcohol dependence in a large population sample from the United States. Arch Gen Psychiatry 59: 825-831.
92. Lagraauw HM, Westra MM, Bot M, Wezel A, van Santbrink PJ, et al. (2014) Vascular neuropeptide Y contributes to atherosclerotic plaque progression and perivascular mast cell activation. Atherosclerosis 235: 196-203.
93. Jaakkola U, Kakko T, Seppala H, Vainio-Jylha E, Vahlberg T, et al. (2010) The Leu7Pro polymorphism of the signal peptide of neuropeptide Y (NPY) gene is associated with increased levels of inflammatory markers preceding vascular complications in patients with type 2 diabetes. Microvasc Res 80: 433-439.
94. Landgraf D, McCarthy MJ, Welsh DK (2014) Circadian clock and stress interactions in the molecular biology of psychiatric disorders. Curr Psychiatry Rep 16: 483.
95. van Zuiden M, Kavelaars A, Geuze E, Olff M, Heijnen CJ (2013) Predicting PTSD: pre-existing vulnerabilities in glucocorticoid-signaling and implications for preventive interventions. Brain Behav Immun 30: 12-21.
96. Bachmann AW, Sedgley TL, Jackson RV, Gibson JN, Young RM, et al. (2005) Glucocorticoid receptor polymorphisms and post-traumatic stress disorder. Psychoneuroendocrinology 30: 297-306.

97. Otte C, Wust S, Zhao S, Pawlikowska L, Kwok PY, et al. (2010) Glucocorticoid receptor gene, low-grade inflammation, and heart failure: the Heart and Soul study. J Clin Endocrinol Metab 95: 2885-2891.
98. Gragnoli C (2014) Hypothesis of the neuroendocrine cortisol pathway gene role in the comorbidity of depression, type 2 diabetes, and metabolic syndrome. Appl Clin Genet 7: 43-53.
99. Andero R, Brothers SP, Jovanovic T, Chen YT, Salah-Uddin H, et al. (2013) Amygdala-dependent fear is regulated by Oprl1 in mice and humans with PTSD. Sci Transl Med 5: 188ra173.
100. Nugent NR, Lally MA, Brown L, Knopik VS, McGeary JE (2012) OPRM1 and diagnosis-related posttraumatic stress disorder in binge-drinking patients living with HIV. AIDS Behav 16: 2171-2180.
101. Dunn EC, Solovieff N, Lowe SR, Gallagher PJ, Chaponis J, et al. (2014) Interaction between genetic variants and exposure to Hurricane Katrina on post-traumatic stress and post-traumatic growth: a prospective analysis of low income adults. J Affect Disord 152-154: 243-249.
102. Surakka I, Isaacs A, Karssen LC, Laurila PP, Middelberg RP, et al. (2011) A genome-wide screen for interactions reveals a new locus on 4p15 modifying the effect of waist-to-hip ratio on total cholesterol. PLoS Genet 7: e1002333.
103. Kuehner C, Huffziger S, Witt SH, Rietschel M (2011) PCLO rs2522833 impacts HPA system activity in healthy young adults. Transl Psychiatry 1: e10.
104. Solovieff N, Roberts AL, Ratanatharathorn A, Haloosim M, De Vivo I, et al. (2014) Genetic association analysis of 300 genes identifies a risk haplotype in SLC18A2 for post-traumatic stress disorder in two independent samples. Neuropsychopharmacology 39: 1872-1879.
105. Ng LL, Sandhu JK, Narayan H, Quinn PA, Squire IB, et al. (2014) Proenkephalin and prognosis after acute myocardial infarction. J Am Coll Cardiol 63: 280-289.
106. Citterio L, Ferrandi M, Delli Carpini S, Simonini M, Kuznetsova T, et al. (2013) cGMP-dependent protein kinase 1 polymorphisms underlie renal sodium handling impairment. Hypertension 62: 1027-1033.
107. Nievergelt CM, Maihofer AX, Mustapic M, Yurgil KA, Schork NJ, et al. (2015) Genomic predictors of combat stress vulnerability and resilience in U.S. Marines: A genome-wide association study across multiple ancestries implicates PRTFDC1 as a potential PTSD gene. Psychoneuroendocrinology 51: 459-471.
108. Zhang C, Wang L, Liao Q, Zhang L, Xu L, et al. (2013) Genetic associations with hypertension: meta-analyses of six candidate genetic variants. Genet Test Mol Biomarkers 17: 736-742.
109. Logue MW, Baldwin C, Guffanti G, Melista E, Wolf EJ, et al. (2013) A genome-wide association study of post-traumatic stress disorder identifies the retinoid-related orphan receptor alpha (RORA) gene as a significant risk locus. Mol Psychiatry 18: 937-942.
110. Guffanti G, Ashley-Koch AE, Roberts AL, Garrett ME, Solovieff N, et al. (2014) No association between RORA polymorphisms and PTSD in two independent samples. Mol Psychiatry 19: 1056-1057.
111. Zhang L, Ursano RJ, Li H (2012) P11: a potential biomarker for posttraumatic stress disorder. Methods Mol Biol 829: 453-468.
112. Huan T, Esko T, Peters MJ, Pilling LC, Schramm K, et al. (2015) A Meta-analysis of Gene Expression Signatures of Blood Pressure and Hypertension. PLoS Genet 11: e1005035.

113. Itokawa K, Sora I, Schindler CW, Itokawa M, Takahashi N, et al. (1999) Heterozygous VMAT2 knockout mice display prolonged QT intervals: possible contributions to sudden death. Brain Res Mol Brain Res 71: 354-357.
114. Zhang J, Sheerin C, Mandel H, Banducci AN, Myrick H, et al. (2014) Variation in SLC1A1 is related to combat-related posttraumatic stress disorder. J Anxiety Disord 28: 902-907.
115. Gressier F, Calati R, Balestri M, Marsano A, Alberti S, et al. (2013) The 5-HTTLPR polymorphism and posttraumatic stress disorder: a meta-analysis. J Trauma Stress 26: 645-653.
116. Grabe HJ, Spitzer C, Schwahn C, Marcinek A, Frahnow A, et al. (2009) Serotonin transporter gene (SLC6A4) promoter polymorphisms and the susceptibility to posttraumatic stress disorder in the general population. Am J Psychiatry 166: 926-933.
117. Moyer AM, Walker DL, Avula R, Lapid MI, Kung S, et al. (2015) Relationship of Genetic Variation in the Serotonin Transporter Gene (SLC6A4) and Congenital and Acquired Cardiovascular Diseases. Genet Test Mol Biomarkers 19: 115-123.
118. Gillespie CF, Almli LM, Smith AK, Bradley B, Kerley K, et al. (2013) Sex dependent influence of a functional polymorphism in steroid 5-alpha-reductase type 2 (SRD5A2) on post-traumatic stress symptoms. Am J Med Genet B Neuropsychiatr Genet 162b: 283-292.
119. Cao C, Wang L, Wang R, Dong C, Qing Y, et al. (2013) Stathmin genotype is associated with reexperiencing symptoms of posttraumatic stress disorder in Chinese earthquake survivors. Prog Neuropsychopharmacol Biol Psychiatry 44: 296-300.
120. Rao F, Zhang K, Zhang L, Rana BK, Wessel J, et al. (2010) Human tyrosine hydroxylase natural allelic variation: influence on autonomic function and hypertension. Cell Mol Neurobiol 30: 1391-1394.
121. Li J, Ding JD, Fang X, Zhang H, Zheng RL, et al. (2012) [Metalloproteinase Tolloid-like 1 gene mutation in Chinese patients with sporadic congenital heart diseases]. Zhonghua Xin Xue Guan Bing Za Zhi 40: 402-405.
122. Vendrell J, Fernandez-Real JM, Gutierrez C, Zamora A, Simon I, et al. (2003) A polymorphism in the promoter of the tumor necrosis factor-alpha gene (-308) is associated with coronary heart disease in type 2 diabetic patients. Atherosclerosis 167: 257-264.
123. Qi L, Zhi J, Zhang T, Cao X, Sun L, et al. (2015) Inhibition of microRNA-25 by tumor necrosis factor alpha is critical in the modulation of vascular smooth muscle cell proliferation. Mol Med Rep.
124. Feldman AM, Combes A, Wagner D, Kadakomi T, Kubota T, et al. (2000) The role of tumor necrosis factor in the pathophysiology of heart failure. J Am Coll Cardiol 35: 537-544.
125. Sesti LF, Crispim D, Canani LH, Polina ER, Rheinheimer J, et al. (2015) The -308G>a polymorphism of the TNF gene is associated with proliferative diabetic retinopathy in Caucasian Brazilians with type 2 diabetes. Invest Ophthalmol Vis Sci 56: 1184-1190.
126. Goenjian AK, Bailey JN, Walling DP, Steinberg AM, Schmidt D, et al. (2012) Association of TPH1, TPH2, and 5HTTLPR with PTSD and depressive symptoms. J Affect Disord 140: 244-252.
127. Li L, Morimoto S, Take S, Zhan DY, Du CK, et al. (2012) Role of brain serotonin dysfunction in the pathophysiology of congestive heart failure. J Mol Cell Cardiol 53: 760-767.

128. Wilker S, Kolassa S, Vogler C, Lingenfelder B, Elbert T, et al. (2013) The role of memory-related gene WWC1 (KIBRA) in lifetime posttraumatic stress disorder: evidence from two independent samples from African conflict regions. Biol Psychiatry 74: 664-671.
